# The Activation of Ti-Zr-V-Hf Non-Evaporable Getter Films with Open-Cell Copper Metal Foam Substrates

**DOI:** 10.3390/ma13204650

**Published:** 2020-10-18

**Authors:** Jie Wang, Jing Zhang, Yong Gao, Yaocheng Hu, Zhiming You, Yupeng Xie, Haipeng Li, Yue Wu, Shanghui Yang, Dengwang Wang, Sheng Wang, Zhanglian Xu

**Affiliations:** 1 Shaanxi Engineering Research Center of Advanced Nuclear Energy & Shaanxi Key Laboratory of Advanced Nuclear Energy and Technology & School of Energy and Power Engineering, Xi’an Jiaotong University, Xi’an 710049, China; wangjie1@xjtu.edu.cn (J.W.); zhangjing1108@stu.xjtu.edu.cn (J.Z.); gaoyong1108@stu.xjtu.edu.cn (Y.G.); hyc1997@stu.xjtu.edu.cn (Y.H.); youzm19960311@stu.xjtu.edu.cn (Z.Y.); xieyupeng@stu.xjtu.edu.cn (Y.X.); lihaipeng@xjtu.edu.cn (H.L.); y574055234@stu.xjtu.edu.cn (S.Y.); wdw21s@163.com (D.W.); 2 Department of Engineering Physics, Tsinghua University, Beijing 100084, China; y-wu20@mails.tsinghua.edu.cn

**Keywords:** accelerators, surface morphology, surface chemical states

## Abstract

Secondary electron emission (SEE) inhibition and vacuum instability are two important issues in accelerators that may induce multiple effects in accelerators, such as power loss and beam lifetime reduction. In order to mitigate SEE and maintain high vacuum simultaneously, open-cell copper metal foam (OCMF) substrates with Ti-Zr-V-Hf non-evaporable getter (NEG) coatings are first proposed, and the properties of surface morphology, surface chemistry and secondary electron yield (SEY) were analyzed for the first time. According to the experimental results tested at 25 °C, the maximum SEY ($\delta_{max}$) of OCMF before and after Ti-Zr-V-Hf NEG film deposition were 1.25 and 1.22, respectively. The XPS spectra indicated chemical state changes of the metal elements (Ti, Zr, V and Hf) of the Ti-Zr-V-Hf NEG films after heating, suggesting that the NEG films can be activated after heating and used as getter pumps.

## 1. Introduction

For the search of possible dark matter (DM) and the study of Higgs physics, several high-energy and high-luminosity accelerators such as the future circular collider (FCC) and the super proton–proton collider (SPPC) have been proposed, with their vacuum systems facing two main challenges of high vacuum gradient and the electron cloud (EC) issues.

On one hand, to reduce the ultimate pressure and vacuum gradient as well as maintain the vacuum stability in accelerators, non-evaporable getter (NEG) films such as Ti-Zr-V films [1,2,3] have been deposited on the inner surface of the vacuum pipes or getter pumps in vacuum systems [2,4], thanks to their distributed pumping properties [3,5,6,7]. On the other hand, to suppress electron clouds in vacuum pipes, various solutions, such as the laser ablation technique [8], artificially grooving surfaces [9], carbon coatings [10] and TiN films [11], etc., have been developed. Generally speaking, previous studies have focused on these two issues separately. A solution for both issues of high vacuum gradient and EC effect has rarely been mentioned before.

NEG films are usually deposited on a flat metal surface. To further improve the residual gas absorption capacity of the film, porous metal substrates with a high contact surface area can be used. Meanwhile, in consideration of the EC effect, the SEY of porous metal substrates can be optimized.

Porous materials [12,13,14] have been developed rapidly over the last decades because of their excellent properties [15,16,17,18], such as large specific surface area [19]. As a porous material, porous metals can be used in electronics and communication [20], energy [21,22], transportation [23,24], bioengineering industries [25,26,27], and so on. With the advantage of porous structures, foam materials can be used as supports for composites by providing a large contact area for the fillers. For example, a film containing ilmenite has been proposed for depositing on foam glass to improve its absorption properties for electromagnetic radiation with high frequency due to its low reflection and light weight [28]. Moreover, Leong et al. introduced and optimized the pore density of aluminum foams to further improve heat transfer in electronic systems, taking advantage of the high thermal conductivity and large contact surface area of metal foams [29].

It has been reported that the Ti-Zr-V-Hf NEG coating has a lower activation temperature of about 150 °C [30]. In this study, open-cell copper metal foam (OCMF) was initially used as the substrate for Ti-Zr-V-Hf NEG coatings to mitigate SEE and provide a good performance of pumping in a vacuum. The structural properties, secondary electron yields (SEYs) and activation process of Ti-Zr-V-Hf NEG films with OCMF substrates were investigated for the first time.

## 2. Experiments and Methods

### 2.1. Characterization Methods

The surface morphologies of the OCMF substrates before and after film deposition were determined using a JEOL 7800F Schottky field scanning electron microscope (SEM, Japan Electron Optics Laboratory, Tokyo, Japan) and Zeiss GeminiSEM 500 emission scanning electron microscope (FE-SEM, Carl Zeiss GmbH, Hallbergmoos, Germany) with an energy dispersive spectrometer (EDS) system to characterize the cross-section and surface morphologies and the distribution maps of related elements. The secondary electron emission properties of the samples were tested using the SEY test equipment. During the SEY tests, the beam diameter and beam current were about 2 mm and 8 nA, respectively. For the SEY and XPS tests, the average heating rates were about 1.2 min/°C at a pressure of about 1 × 10^−7^ Pa. Surface compositions tests were carried out with an X-ray photoelectron spectroscope (Thermo Fisher ESCALAB Xi^+^, Thermo Fisher Scientific, Waltham, MA, USA), which was calibrated with a binding energy of C1s peak of 284.8 eV.

### 2.2. Sample Preparation 

The OCMF substrates with the dimension of 10 mm × 10 mm × 1 mm were purchased from Kunshan Longshengbao Electronic Materials Co., Ltd. (Kunshan, China) and prepared by the metal deposition method. The copper was deposited on the open-cell polymer frameworks, followed by polymer elimination and materials sintering.

**Figure 1:**
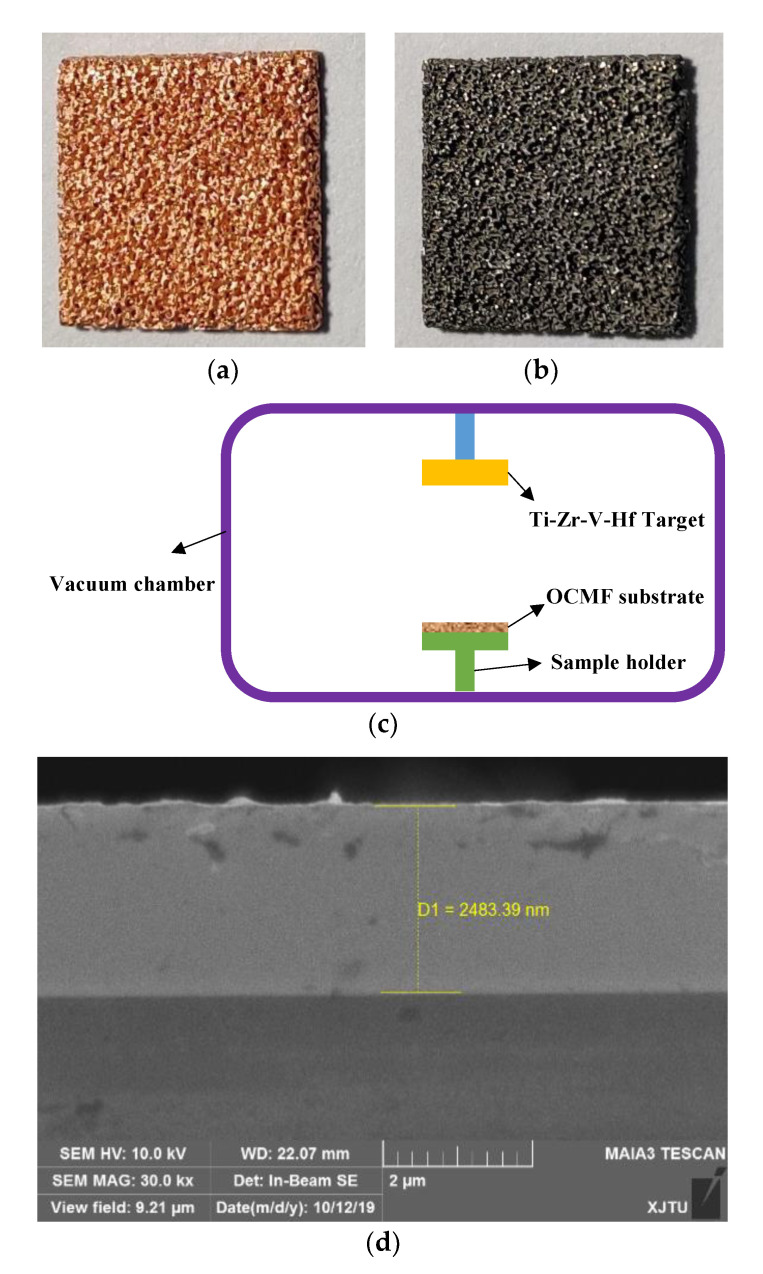
The photos of the open-cell copper metal foam (OCMF) substrates with the pore size of 0.1 mm (**a**) before and (**b**) after film deposition. (**c**) The schematic of the DC sputtering deposition machine. (**d**) The SEM cross-section images of the Ti-Zr-V-Hf film on silicon substrates.

Before the Ti-Zr-V-Hf NEG coating deposition, the OCMF substrates with a pore size of 0.1 mm were ultrasonically rinsed in acetone and then in absolute ethyl alcohol for 10 min, respectively. Then the TiHf film coatings were deposited on the OCMF substrates by DC sputtering using Ar gas with a purity of 99.999%. The target material was made of Ti, Zr, V and Hf elements with an atomic number ratio of 1:1:1:1. Typical film deposition parameters were a working pressure of 0.5 Pa, a discharge power of 280 W, a gas flow of 20 Sccm, a sputtering rate of 1.39 nm/s and a base pressure of 5.8 × 10^−4^ Pa. The temperature of the sample disk increased from 25 °C to 40 °C during Ti-Zr-V-Hf non-evaporable getter film deposition. Three Ti-Zr-V-Hf non-evaporable getter films with open-cell copper metal foam substrate samples prepared by the same film deposition parameters were used for the same tests, such as the SEM tests. The photos of the OCMF substrates before and after film deposition are shown in Figure 1a,b. The model of the DC sputtering deposition machine is shown schematically in Figure 1c. Silicon wafers were used to evaluate the thickness of the Ti-Zr-V-Hf NEG coatings on the porous OCMF substrates, which was 2.48 μm, as shown in Figure 1d.

## 3. Results and Discussion

### 3.1. Surface Morphology

**Figure 2:**
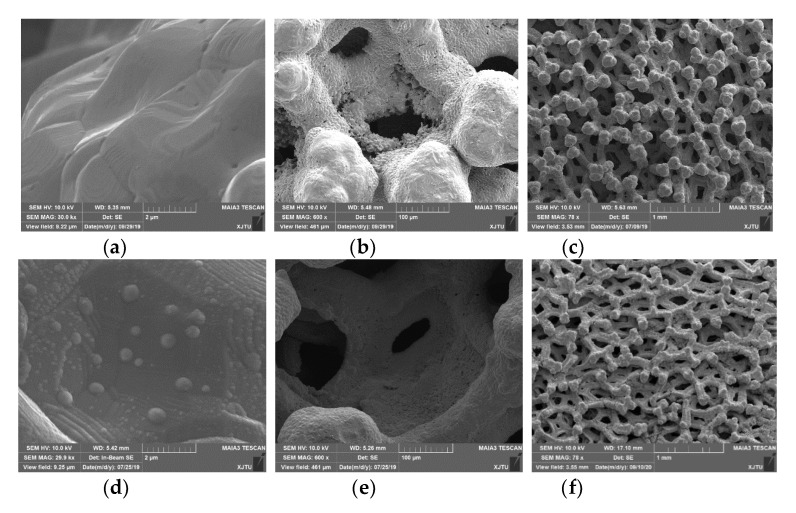
The SEM images of the OCMF substrates and Ti-Zr-V-Hf-coated OCMF. (**a**), (**b**) and (**c**) are the surface morphologies of the OCMF substrates with different magnifications of 30,000×, 600× and 78×. (**d**), (**e**) and (**f**) are the surface morphologies of Ti-Zr-V-Hf-coated OCMF substrates with different magnifications of 30,000×, 600× and 78×.

The surface morphologies of OCMF substrates with a pore size of 0.1 mm are shown in Figure 2a–c with different magnifications. The wave-like structures, as shown in Figure 2a, appeared on the surface of the OCMF substrates. At the same time, island-like structures formed on the surface of the Ti-Zr-V-Hf film-coated OCMF substrates, as manifested in Figure 2d with the SEM image magnifications of 30,000×, which may have induced the roughness increase compared to that of the uncoated OCMF substrates in Figure 2a.

**Figure 3:**
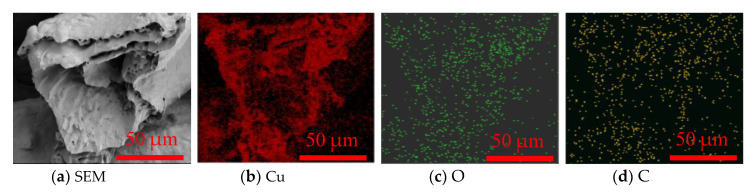
The cross-section SEM images of the OCMF substrates (**a**) with the corresponding elemental distribution maps for Cu (**b**), O (**c**) and C (**d**).

To analyze the element distributions of the OCMF substrates before and after film deposition, SEM and EDS analyses were performed. A cross-section SEM image of the OCMF substrates is shown in Figure 3a. The EDS results indicated the existence of three elements (Cu, C and O) on the cross-section of the OCMF substrates (Figure 3b–d). This may indicate that the OCMF substrate surface was partially oxidized during air exposure.

**Figure 4:**
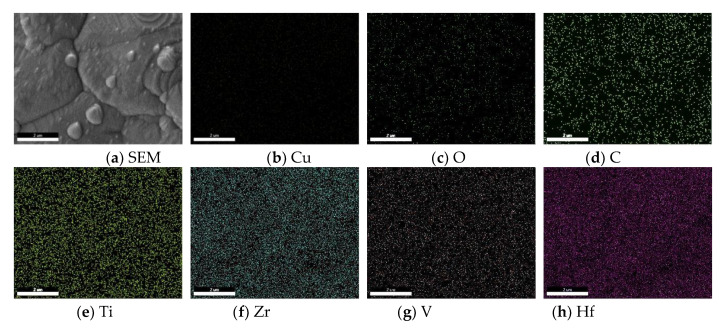
The surface SEM images of the Ti-Zr-V-Hf film with OCMF substrates (**a**) and corresponding Cu (**b**), O (**c**), C (**d**), Ti (**e**), Zr (**f**), V (**g**) and Hf (**h**) distribution maps. Scale bar 2 μm.

The surface EDS analysis was conducted to analyze the element distributions of the Ti-Zr-V-Hf film shown in Figure 4a, with relevant maps shown in Figure 4b–h. It can be seen that Ti, Zr, V and Hf elements had a uniform distribution, while the Cu element was not found on the surface. This may indicate that the OCMF substrate surface was basically fully covered by the Ti-Zr-V-Hf NEG film. A small quantity of the element O was detected, probably due to the air exposure during sample transfer, as shown in Figure 4c. Moreover, the elements of C and O may have been contributed by adventitious carbon or hydrocarbons.

### 3.2. SEY

**Figure 5:**
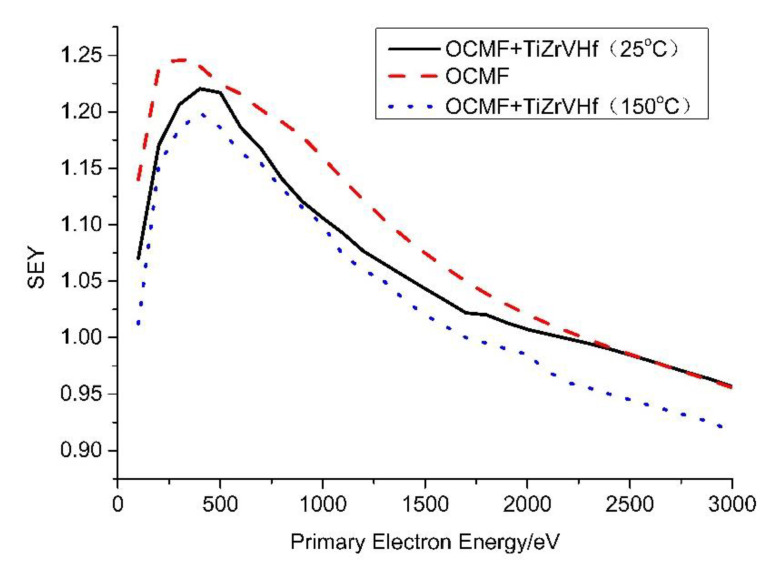
Secondary electron yield (SEY) curves of OCMF substrates and Ti-Zr-V-Hf film-coated OCMF tested at 25 °C and 150 °C.

As shown in Figure 5, the $\delta_{max}$ of the OCMF substrates before and after Ti-Zr-V-Hf film deposition with a thickness of 2.48 μm were 1.25 and 1.22 (tested at 25 °C), with a corresponding primary electron energy of 300 eV and 500 eV, respectively. The $\delta_{max}$ of Ti-Zr-V-Hf film with OCMF substrates after heating in a vacuum (at 100 °C for 2 h and then 150 °C for 2 h) was 1.20. It can be seen that the $\delta_{max}$ of the coated OCMF substrates decreased about 0.03, compared to that of the uncoated one. Under the condition of heating in a vacuum, the $\delta_{max}$ of Ti-Zr-V-Hf-coated OCMF further decreased to 1.20, which may be ascribed to surface chemical state changes during the heating process.

As reported by Baglin et al. [31,32], the $\delta_{max}$ of flat copper was about 1.9–2.5. Compared to the $\delta_{max}$ of flat copper, that of the OCMF substrates decreased about 0.65–1.25. The electron cloud threshold for large hadron collider (LHC) arcs and SPPC are $\delta_{max}$ < 1.5 and $\delta_{max}$ < 1.2, respectively [33,34]. Therefore, OCMF coated with Ti-Zr-V-Hf NEG film can be considered for application in the vacuum systems of accelerators. However, the $\delta_{max}$ less than 1 would be better for electron cloud mitigation and power dissipation reduction in the cryogenic region of the vacuum chambers.

### 3.3. Surface Chemical States Variation during Activation

**Figure 6:**
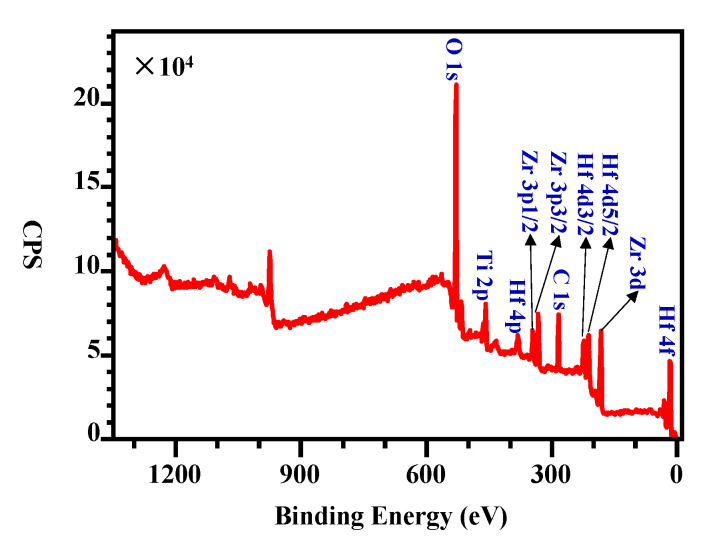
The XPS survey scan of the Ti-Zr-V-Hf film at 25 °C.

**Figure 7:**
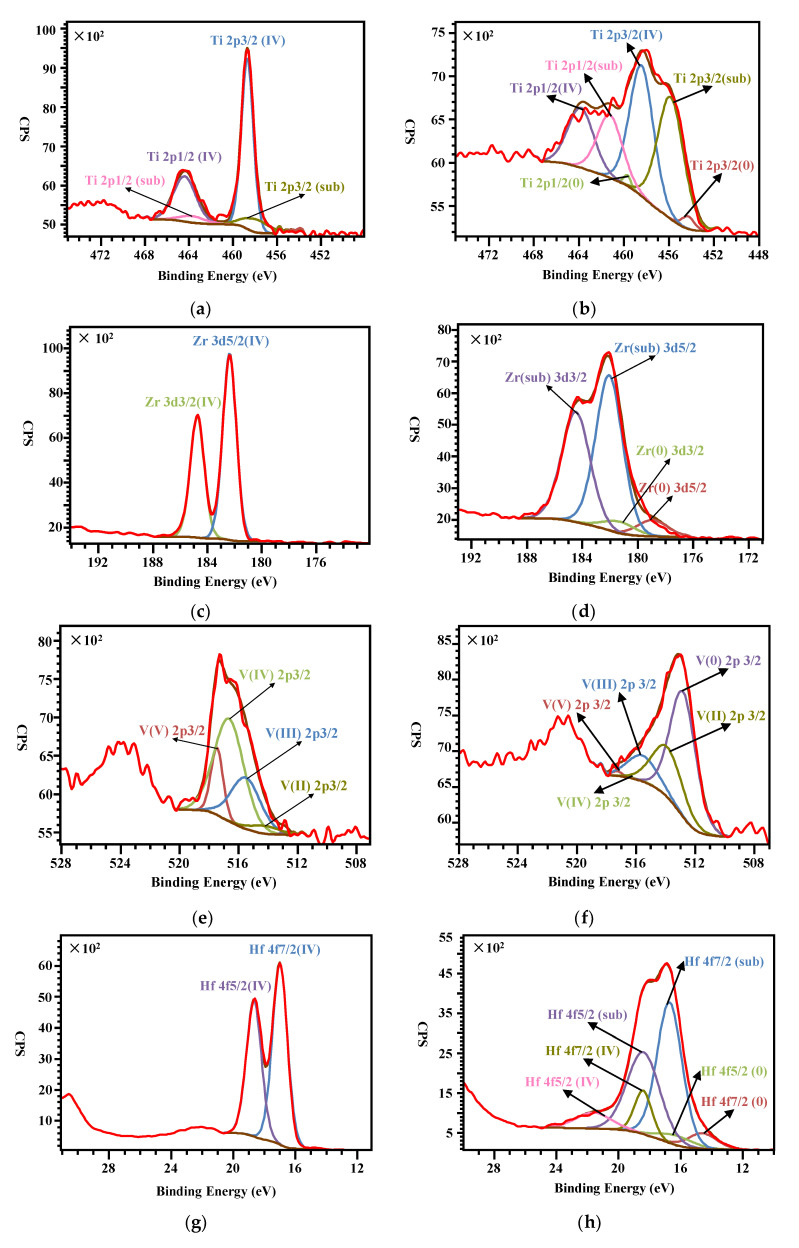
The XPS energy spectra of (**a**) Ti, (**c**) Zr, (**e**) V and (**g**) Hf tested at 25 °C and (**b**) Ti, (**d**) Zr, (**f**) V and (**h**) Hf tested at 150 °C of the Ti-Zr-V-Hf film with OCMF substrates, respectively.

According to the XPS survey scan of the Ti-Zr-V-Hf film tested at 25 °C shown in Figure 6, it has a metal element ratio of 2.3 (Ti):2.7 (Zr):1.0 (V):1.7 (Hf). After exposure in air, the NEG films need to be reactivated after heating treatment at a certain temperature in an ultrahigh vacuum to recover the pumping ability. During the NEG film activation process, the oxygen present in the surface passivation layer is mainly diffused into the vacuum and deep inside the film. For the activation of Ti-Zr-V-Hf NEG film with OCMF substrates, it was heated at 100 °C for 2 h, and then at 150 °C for 2 h during the XPS tests. The binding energy spectra of Ti, Zr, V and Hf elements of the film sample tested at 25 °C and then at 150 °C are shown in Figure 7a–h. During NEG film activation, the Ti 2p, Zr 3d, V 2p and Hf 4f peak shifts occurred. The XPS spectra showed distinct variations when the Ti-Zr-V-Hf NEG film surface became activated after heating (see Figure 7).

The high-resolution photoelectron spectrum of Ti in Figure 7a indicates that Ti metal was basically fully oxidized before heating, which was mainly composed of titanium sub-oxide (Ti-sub) and titanium oxide (Ti^4+^) with the concentrations of 84.5 at% and 13.8 at%. According to the previous results, the components of 454.0 eV [35,36,37] and 458.7 eV [38,39,40] can attribute to the Ti metal and Ti^4+^, respectively. However, after being activated at 150 °C, the Ti metal appeared to have a concentration of 2.41%. Moreover, the content ratio of Ti^4+^ decreased progressively from 84.5 at% to 48.6 at%, and that of Ti-sub increased from 13.8 at% to 49.1 at%, as shown in Figure 7a,b.

Figure 7c,d illustrates the Zr 3d peaks of the Ti-Zr-V-Hf film before and after heating, respectively. The analysis of these two spectra indicates that: (1) the peaks at 182.4 eV and 184.7 eV in Figure 7c can be ascribed to the state of zirconium oxide (Zr^4+^) [41]. Based on the spectrum of Zr tested at 25 °C, the Zr metal on the surface of the Ti-Zr-V-Hf film was basically fully oxidized. (2) At the same time, the zirconium sub-oxide and zirconium metal appeared after heating, with the content ratios of 89.4 at% and 10.6%, respectively. The heating for the Ti-Zr-V-Hf NEG film activation resulted in the oxidation state change of zirconium.

The vanadium XPS spectrum of Figure 7e tested at 25 °C demonstrate that the V 2p were associated with V(0), V(II), V(III), V(IV) and V(V), with the corresponding content ratios of 0.5 at%, 4.1 at%, 27.0 at%, 54.0 at% and 14.4 at%, respectively. Nevertheless, the vanadium XPS spectrum after heating tested at 150 °C, is composed of V(0), V(II), V(III), V(IV) and V(V) oxide multiplet peaks with the atom ratios of 57.5 at%, 27.1 at%, 14.0 at%, 0.5 at% and 0.9 at%, respectively, as shown in Figure 7f. It can be seen that the atom ratios of V(0) and V(II) increased significantly, and those of V(III), V(IV), and V(V) decreased distinctly during the Ti-Zr-V-Hf NEG film activation.

The interpretation of the Hf 4f XPS spectra in Figure 7g,h was to determine the content ratios of the Hf(0), Hf(sub) and Hf(IV) oxidation states to understand the activation process. The fitting results of the Hf 4f XPS spectrum tested at 25 °C in Figure 7g indicate that the Hf metal was basically oxidized on the surface of the Ti-Zr-V-Hf NEG film. The binding energy of Hf(IV) 4f7/2 is 17.0 eV, which is in accordance with the reported references [42,43]. Whereas the spectrum of Hf 4f XPS spectrum tested at 150 °C shown in Figure 7h demonstrates that the Hf metal, Hf suboxide and Hf oxide appeared with the percentages of 9.4 at%, 74.8 at% and 15.8 at%, respectively.

## 4. Conclusions

The OCMF material was first proposed in this paper to inhibit the SEE in accelerators or vacuum devices, etc. In addition, Ti-Zr-V-Hf NEG films were deposited on OCMF substrates for the first time to improve the pumping properties in a vacuum system.

First, the SEYs of the OCMF substrates before and after Ti-Zr-V-Hf NEG film deposition were evaluated by the SEY measurements. The SEY results indicate that OCMF is a very promising solution for reducing the $\delta_{max}$ to below 1.25. After Ti-Zr-V-Hf NEG film deposition on the OCMF substrates, the $\delta_{max}$ further decreased to 1.22, and then to 1.20, following heating in a vacuum.

Second, OCMFs coated with a NEG film can also act as getter pumps to improve the vacuum. The analysis of the chemical states of Ti, Zr, V and Hf NEG films indicated a distinct valence state and atomic ratio changes during activation.

Finally, the related experimental results mentioned above demonstrate that the Ti-Zr-V-Hf NEG film deposited on the OCMF substrates can be activated and used as the getter pumps in the vacuum, also with the advantage of low SEY properties. With the advantages of low density and large contact areas, OCMF can be used for electron cloud mitigation and maintaining a high vacuum in accelerators or high-power microwave devices, etc.
